# *Nebula*: reconstruction and visualization of scattering data in reciprocal space

**DOI:** 10.1107/S1600576715001788

**Published:** 2015-02-21

**Authors:** Andreas Reiten, Dmitry Chernyshov, Ragnvald H. Mathiesen

**Affiliations:** a Department of Physics, Norwegian University of Science and Technology, Trondheim, Norway; b Swiss–Norwegian Beamlines, European Synchrotron Radiation Facility, Grenoble, France

**Keywords:** diffuse scattering, data analysis and visualization, computer programs

## Abstract

Two-dimensional solid-state X-ray detectors can now operate at considerable data throughput rates that allow full three-dimensional sampling of scattering data from extended volumes of reciprocal space within second to minute time­scales. For such experiments, simultaneous analysis and visualization allows for remeasurements and a more dynamic measurement strategy. A new software, *Nebula*, is presented. It efficiently reconstructs X-ray scattering data, generates three-dimensional reciprocal space data sets that can be visualized interactively, and aims to enable real-time processing in high-throughput measurements by employing parallel computing on commodity hardware.

## Abstract 2

A software to reconstruct and visualize diffuse scattering in three-dimensional reciprocal space using data from X-ray area detectors is presented.

## 1. Terminology   

Texel, texture element or texture pixel: the fundamental unit of texture space used in computer graphics. Textures are represented by arrays of texels, just as pictures are represented by arrays of pixels.

Viewing frustum: in three-dimensional computer graphics, the region of space in the modeled world that may appear on the screen; it is the field of view of the notional camera.

Graphics processing unit (GPU): a specialized electronic circuit designed to rapidly manipulate and alter memory to accelerate the creation of images in a frame buffer intended for output to a display.

Memory bandwidth: the rate at which data can be read from or stored into a semiconductor memory by a processor.

OpenGL (Open Graphics Library): a cross-language multi-platform application programming interface for rendering two-dimensional and three-dimensional vector graphics (http://opengl.org).

OpenCL (Open Computing Language): a framework for writing programs that execute across heterogeneous platforms consisting of central processing units, graphics processing units and other processors (http://khronos.org/opencl).

Qt: a cross-platform application framework that is widely used for developing application software with a graphical user interface (http://qt-project.org).

Git: a distributed revision control and source code management system (http://git-scm.com).

## 2. Introduction   

With the emergence of fast two-dimensional solid-state X-ray detectors (Kraft *et al.*, 2009 ▶), full three-dimensional sampling of scattering data from extended volumes of reciprocal space may be carried out within second to minute timescales, provided that the X-ray source is adequately bright. This is particularly useful in experiments that seek to investigate the volumetric properties of features in reciprocal space, *e.g.* diffuse scattering, finite size effects, multiple twins or crystallites, *etc*. Faster data acquisition also opens up opportunities for novel studies where material response may be investigated *in situ* during external loading, with, for example, diffuse scattering as the response probe. For such three-dimensional scattering experiments, simultaneous analysis and visualization is a major advantage, allowing for remeasurements in the event of flaws and on-the-fly reassessment of measurement strategy.

There are currently a few programs available that specialize in visualizing three-dimensional volumes. *Max3D* (Britten & Guan, 2007 ▶; http://www.chemistry.mcmaster.ca/facilities/xray/221-max3d) resamples data onto a voxel grid and lets the user manually reload smaller diffraction volumes at higher resolution. *Chimera* (Pettersen *et al.*, 2004 ▶) is an extensible program for interactive visualization and analysis of molecular structures and related data that can also be used for reciprocal space viewing. Like *Max3D* it uses voxel grids for volume viewing, but it is not concerned with data reconstruction. Falch *et al.* (2013 ▶) describe a visualization method that distinguishes itself by operating directly on the unstructured samples, rather than resampling them to form voxels. They also employ an octree data structure to achieve faster rendering.

The software presented here works by resampling data to form a voxel octree and can be seen as a hybrid between the approaches taken in *Max3D* and by Falch *et al.* (2013 ▶). The resampling combined with the octree structure help improve the rendering time and allow the data to span over large regions. The construction of the octree is also relatively fast, as heavy computations are done on the GPU where applicable. The aim of the program is rapid reconstruction and interactive visualization of three-dimensional data, meeting real-time processing requirements of high-throughput measurements.

## 3. Program specification   

The software is divided into two major modules unified by a graphical user interface. The first module administers file selection and reconstruction, including generation of three-dimensional data sets. The second module handles volume rendering of the data sets and is equipped with a toolkit to help users view and analyze features. This section describes the workings of the two modules, and continues to specify features, performance, and the software and hardware environments.

### 3.1. Reconstruction   

The reconstruction procedure by which two-dimensional X-ray images are projected onto the Ewald sphere can be divided into three steps. First, the real-space position of each image pixel is calculated using the detector and diffractometer geometry found in the image headers. The program reads CBF files (Bernstein & Hammersley, 2005 ▶) and assumes a four-circle κ diffractometer (Thorkildsen *et al.*, 1999 ▶). Other geometries and file format interpreters can be implemented on demand. The pixel position relative to the sample dictates the direction of the scattered ray and allows the reciprocal space vector, 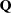, to be expressed as 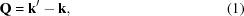
where 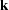 denotes the wavevector of the incident beam and 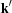 is the wavevector of the scattered ray. 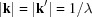 under the kinematic approximation, where λ is the wavelength.

**Figure 1:**
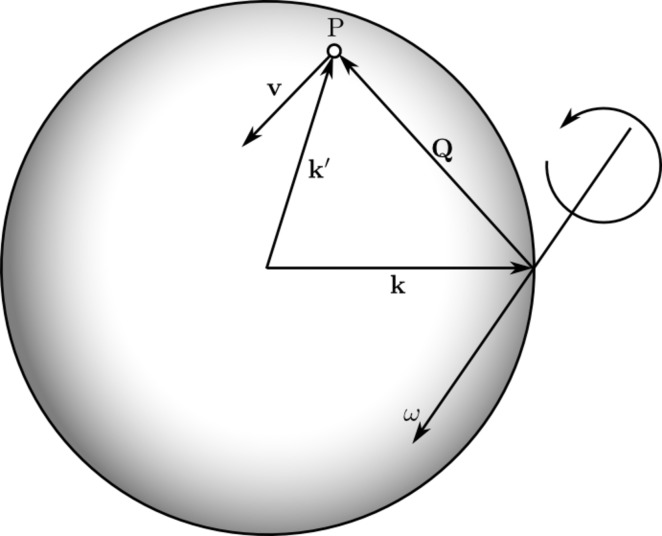
The Lorentz correction is governed by the orientation of the sample rotation axis, given by the angular velocity vector 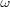, with respect to the scattering vector 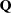. The point P represents a reciprocal lattice node in the instant it rotates through and intersects with the Ewald sphere. Specifically, the correction is proportional to the velocity vector 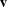 of the point P projected onto the unit wavevector 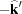 of the scattered ray.

Second, a set of corrections are applied to the intensity value of each image pixel. These include a Lorentz–polarization correction, incident beam flux correction and background subtraction. The Lorentz correction is given by
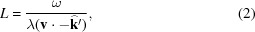
where 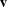 is the velocity of a reciprocal node as it is rotated through and intersects with the Ewald sphere at angular speed ω, as shown in Fig. 1 ▶ (Buerger, 1940 ▶). The velocity vector is defined by the sample rotation axis and 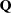. The polarization correction is given by 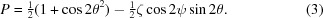
Here θ is the Bragg angle, ψ is the angle between the diffracting plane and the plane of incidence, and
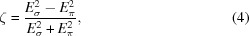
where 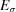 is the amplitude of the σ-polarized field, *i.e.* the component oscillating in the plane of incidence, and 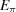 is the amplitude of the component oscillating perpendicular to it (Kahn *et al.*, 1982 ▶).

The background subtraction is approximated for each frame by a linear least-squares fit of a plane through a number of user-defined pixel regions that are deemed representative of the background. The regions can be selected for each frame individually or for a series of frames. The maximum intensity for each pixel position in a series can be found and visualized to help distinguish regions that remain background throughout the scan. Optionally, a uniform background value can be subtracted for simplicity. The latter method can be used to quickly yield a data set of manageable size suitable for a first inspection in three dimensions. A more rigorous and yet universally applicable background correction is currently beyond the scope of the software.

Third, data above zero intensity are put into an octree structure to facilitate interpolation. The data are then sampled over several overlapping voxel grids, each one with twice the resolution of the last, such that each voxel is repeatedly divided into eight new blocks until a desired resolution has been reached. Since only non-empty blocks of a grid are sampled further, the result is a sparse data structure called a sparse voxel octree (Crassin *et al.*, 2009 ▶; Gobbetti *et al.*, 2008 ▶) (*cf*. Fig. 3 below).

### 3.2. Visualization   

**Figure 2:**
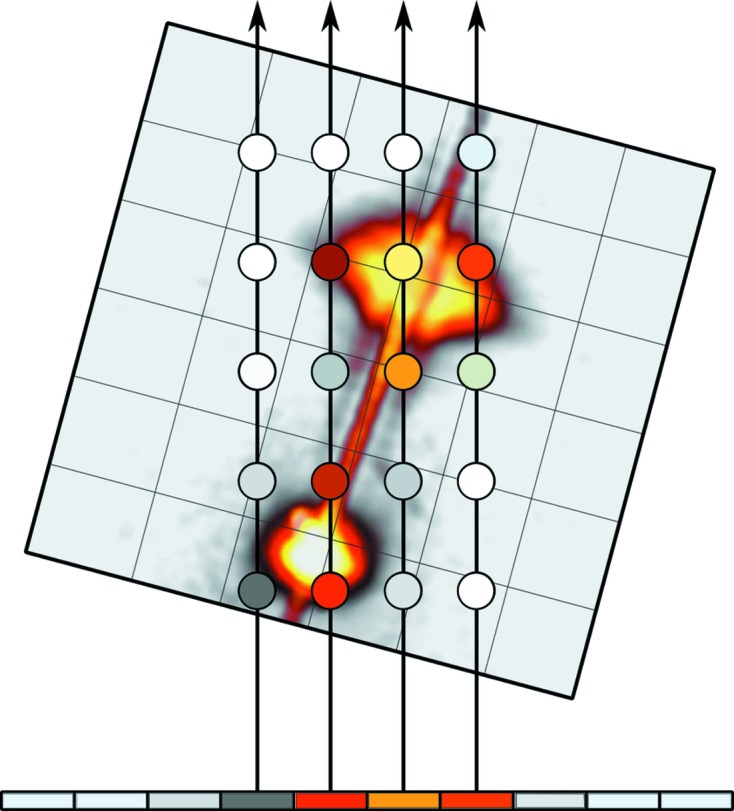
Volume ray casting is a rendering technique in which rays traverse and sample a volume. Here the view plane is depicted from above.

The sparse data sets are visualized using volume ray casting (Levoy, 1988 ▶; Drebin *et al.*, 1988 ▶). In short, each texel on the screen used for rendering corresponds to a ray penetrating the volume containing the data set. The direction and extent of each ray is determined by a viewing frustum extending from a hypothetical camera point. The rays traverse the data and samples are taken along each ray in a front-to-back manner, with each intensity sample matched to an RGBA color value. The colors are blended together, and the texel takes the value of the accumulated color upon completing the ray traversal (*cf*. Figs. 2 ▶ and 3 ▶). This mode of visualization is the default and is referred to as normal color blending.

The visualization greatly depends on the choice of transfer function, *i.e.* the RGBA specter to which intensity values are matched. For example, iso-surface extraction can be achieved by assigning nonzero opacity only to a select intensity range, while a transfer function where the alpha level gradually increases with intensity typically will yield partially transparent features.

**Figure 3:**
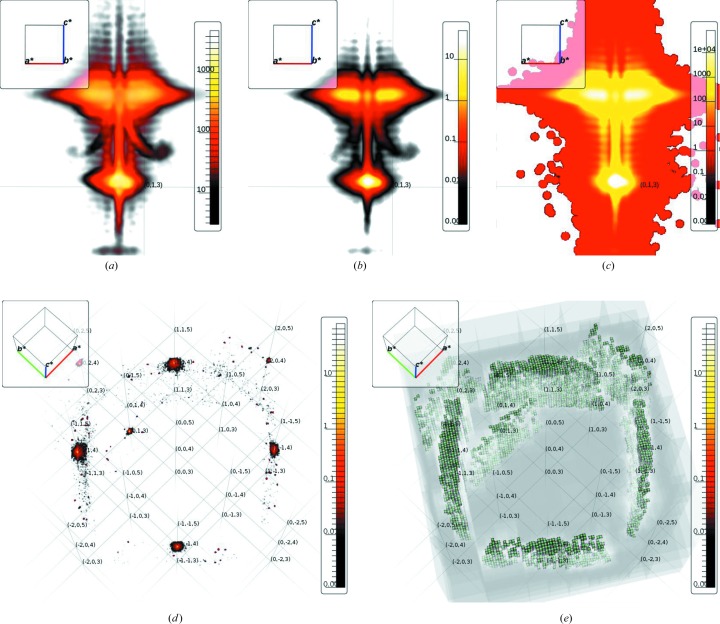
Example reconstruction based on data from an La
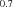
Sr
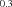
MnO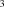 thin film grown along 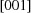 on an SrTiO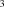 substrate. The diffuse scattering around the 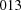 Bragg peak is shown in (*a*) normal color blending, (*b*) integration and (*c*) slice mode. The lower peaks originate from the substrate and the upper features from the thin film. The two blobs left and right of the thin-film peak can be attributed to the two major domain configurations. The positions of the features are in agreement with previous findings (Boschker *et al.*, 2013 ▶). Thickness fringes are easily recognized. (*d*) A zoomed out view of the data set, showing diffuse features superimposed on the corresponding cubic lattice. (*e*) Same as (*d*), but showing the sparse voxel octree structure.

There are in addition two more imaging modes to choose from. The integration mode replaces the normal color blending scheme by a method that integrates sample values along the ray and assigns color corresponding to the final value. This mode requires only minor configuration of the view parameters and is suited to give a first impression of the data. Finally, the slice mode can be used to visualize any cut through the data set. The different modes are shown in Fig. 3 ▶.

### 3.3. Features   

The software has a graphical user interface and a set of basic features. The interface lets the user select files on the hard drive for processing and gives control over essential reconstruction and visualization parameters. A built-in image viewer can be used to inspect files one by one and to remove, for example, overexposed frames prior to reconstruction. The intensity in a series of frames can also be integrated in a specific area to quantitatively compare the relative intensities of features.

A unit-cell overlay can be specified based on a user-provided UB matrix (Busing & Levy, 1967 ▶) and rotated to coincide with the data. It is also possible to visualize custom three-dimensional functions where the input parameters can be changed interactively.

### 3.4. Performance   

The bottleneck for three-dimensional visualization algorithms is computer processing power, which has increased rapidly with continued advances in lithography. Fully capable hardware can now be acquired off the shelf. Notably, this software benefits greatly from using a dedicated graphics processing unit – a common component in most modern computers. This enables the use of OpenCL to parallelize tasks that would otherwise be considerable bottlenecks in the program work flow. In particular, parts of the reconstruction and volume rendering algorithms benefit greatly from parallelization. Consequently, the software requires an OpenCL capable graphics card with sufficient video memory to store data sets during rendering.

During reconstruction, the most time-consuming task is typically to read and decompress data from the hard drive. This step is mainly limited by the CPU and the read speed of the hard drive. Building the sparse voxel octree can be fast in comparison, depending on how much of the raw data set has been omitted, and is limited by CPU speed and graphics card memory bandwidth. Typical run times for the test systems given in Table 1 ▶ are shown in Table 2 ▶.

From a programming standpoint the quality of the visualization is governed by the dimensions of the display texture. Its dimensions can be changed on demand, but in practice there is no gain from increasing it beyond the pixel dimensions it occupies on the screen. Reducing the texture resolution will result in a higher viewing frame rate, and using the test systems in Table 1 ▶ this is often necessary for smooth real-time interaction. The frame rate is limited mainly by the graphics card memory bandwidth.

### 3.5. Software and hardware environment   

The program is written in C++ and uses OpenGL and OpenCL for rendering and parallelized computations, respectively. The graphical user interface is provided by Qt 5. The software has been tested under 64 bit Arch Linux and 64 bit Windows 7, with the hardware specifications as shown in Table 1 ▶. The minimum hardware requirement is an OpenCL 1.1 and OpenGL 4.0 capable graphics card and 2 GB of system RAM. The maximum data set size is limited by the available system RAM, the graphics card RAM and the system hard drive space, but there are no fixed lower bounds on the latter two.

### 3.6. Availability and documentation   

The source code is available under the GNU General Public License at https://github.com/Natnux/nebula. Documentation for users exists in the wiki on the same page. Anyone is invited to browse the source code, and code contributions through Git are greatly appreciated.

## Figures and Tables

**Table 1:** Specification of test systems

	Laptop	Desktop
RAM	16 GB @ 1333MHz	16 GB @ 2400MHz
CPU	Intel Core i7-2630QM @ 2.0GHz	Intel Core i7-4790 @ 3.6GHz
Graphics card	Nvidia GT 560M @ 60.0GBs^1^	Nvidia GTX 760 @ 192.3GBs^1^

**Table 2:** Example run times processing the 1726 frames (4.3GB compressed data) that constitute Fig. 3 ▶ The sparse voxel octree that was generated was 13 levels deep.

System	Reading and reducing data (s)	Octree generation (s)
Laptop, Arch Linux	172	26
Laptop, Windows 7	211	85
Desktop, Arch Linux	117	24
Desktop, Windows 7	127	32
